# Bedtime Snack Composition and Overnight Glycemic Profiles in Children and Adolescents with Type 1 Diabetes Using Multiple Daily Injections: A Randomized Crossover Study

**DOI:** 10.3390/nu18172924

**Published:** 2026-09-07

**Authors:** Didem Güneş Kaya, Elvan Bayramoğlu, Hande Turan, Olcay Evliyaoğlu, Murat Bas

**Affiliations:** 1 Department of Pediatrics, Cerrahpaşa Faculty of Medicine, Istanbul University-Cerrahpaşa, Istanbul 34098, Türkiye; didemguneskaya@iuc.edu.tr; 2 Department of Nutrition and Dietetics, Graduate School of Health Sciences, Acibadem Mehmet Ali Aydinlar University, Istanbul 34638, Türkiye; 3 Department of Pediatric Endocrinology, Cerrahpaşa Faculty of Medicine, Istanbul University-Cerrahpaşa, Istanbul 34098, Türkiye; 4 Department of Nutrition and Dietetics, Faculty of Health Sciences, Acibadem Mehmet Ali Aydinlar University, Istanbul 34638, Türkiye

**Keywords:** type 1 diabetes, bedtime snack, nocturnal glycemia, continuous glucose monitoring, time in range, glycemic variability, macronutrient composition, children, adolescents, multiple daily injections

## Abstract

**Background/Objectives:** Bedtime snacks are frequently used in youth with type 1 diabetes (T1D) to reduce nocturnal hypoglycemia risk; however, the optimal macronutrient composition remains uncertain, particularly in those treated with multiple daily injections (MDI). This randomized crossover study evaluated whether standardized whole-food bedtime snacks with different macronutrient profiles yield distinct overnight continuous glucose monitoring (CGM) patterns. **Methods:** Twelve children and adolescents with T1D using glargine/lispro-based MDI completed four bedtime snack protocols: carbohydrate-only (CHO), carbohydrate-plus-protein (CHO + PRO), carbohydrate-plus-fat (CHO + FAT), and carbohydrate-plus-fiber (CHO + FIB). Each snack was consumed for three consecutive nights at 21:00 without additional bolus insulin. Blinded CGM data were analyzed for 21:00–09:00 and for predefined postprandial intervals. Because no a priori sample size calculation was performed, the study was conducted as an exploratory pilot trial. Nights per snack were averaged before within-subject comparisons. **Results:** Overnight time in range (TIR; 70–180 mg/dL) varied across protocols (*p* = 0.014), with the highest TIR following CHO + PRO (85.8% ± 12.1%) and the lowest following CHO + FAT (70.9% ± 19.1%); however, adjusted whole-night pairwise comparisons were not statistically significant. Differences were more pronounced during the first 6 h following snack consumption, when CHO + PRO was associated with higher TIR than CHO + FAT (91.2% ± 7.6% vs. 71.4% ± 20.8%; adjusted *p* = 0.011). Moreover, CHO + PRO was associated with lower peak-to-nadir glucose amplitude than CHO + FAT (88.1 ± 24.9 vs. 123.9 ± 36.5 mg/dL; adjusted *p* = 0.041). Hypoglycemic and hyperglycemic event rates did not significantly differ across protocols. **Conclusions:** In children and adolescents with T1D using MDI, bedtime snack composition was associated with differences in early nocturnal glycemic stability. CHO + PRO demonstrated higher early night TIR and lower glucose amplitude than CHO + FAT; however, event-based safety outcomes did not significantly differ. Because only two pairwise comparisons remained significant after correction for multiple testing and the four snacks were not matched for energy content, these findings should be regarded as preliminary and hypothesis-generating; in particular, they do not demonstrate that any snack composition protects against nocturnal hypoglycemia. Confirmation is required in larger, adequately powered studies that include energy-matched snacks and a no-snack control condition.

## 1. Introduction

Type 1 diabetes (T1D) management in children and adolescents necessitates achieving glycemic targets while mitigating hypoglycemia risk and excessive treatment burden [1]. Current intensive insulin therapy strategies mainly encompass multiple daily injections (MDI) and continuous subcutaneous insulin infusion (CSII; insulin pump therapy) [2]. However, unlike automated insulin delivery (AID) systems, MDI regimens do not automate insulin delivery reduction or suspension during periods of falling glucose levels overnight [3]. Therefore, the safe management of nocturnal glycemia remains a crucial clinical challenge, particularly in MDI-treated children and adolescents.

In youth with T1D, nocturnal hypoglycemia remains a clinically significant concern because episodes may be prolonged and asymptomatic during sleep [4]. Moreover, fear of nocturnal hypoglycemia may impact children and caregivers, potentially influencing sleep quality, nighttime glucose monitoring practices, and feeding behaviors before bedtime [5]. With the increasing use of continuous glucose monitoring (CGM), the assessment of glycemic management has expanded beyond glycated hemoglobin A1c (HbA1c), which reflects mean glycemia over the preceding 2–3 months but conveys no information on within-day glucose fluctuations, to encompass CGM-derived metrics. These metrics include time in range (TIR), defined as the percentage of time spent between 70 and 180 mg/dL; time below range (TBR), the percentage of time below 70 mg/dL (with values below 54 mg/dL denoting clinically significant, level 2 hypoglycemia); time above range (TAR), the percentage of time above 180 mg/dL (with values above 250 mg/dL denoting level 2 hyperglycemia); and glycemic variability (GV), most commonly expressed as the coefficient of variation (%CV) of sensor glucose values, for which a threshold of 36% distinguishes stable from unstable glucose profiles [1,6]. In particular, CGM facilitates a more comprehensive evaluation of overnight glucose excursions and otherwise unrecognized nocturnal hypoglycemic events [6].

In clinical practice, bedtime snack consumption has long been used as a strategy for mitigating the risk of nocturnal hypoglycemia; however, the effectiveness and necessity of this approach in individuals using modern basal insulin analogs remain uncertain [7,8,9]. Most previous studies were limited by older insulin regimens, small sample sizes, or a lack of CGM-based assessment [7,8]. Available studies have mainly centered on specific nutritional components, including high-fat, high-fiber, low-glycemic-index, or uncooked cornstarch-containing bedtime snacks [10,11,12]. Nevertheless, data comparing the effects of standardized whole-food bedtime snacks with different macronutrient compositions on CGM-derived nocturnal glycemic metrics in MDI-treated children and adolescents remain scarce. Therefore, the optimal composition of bedtime snacks and their effects on nocturnal glycemia in this population remain unclear.

This study aimed to evaluate the effects of standardized whole-food bedtime snacks with different macronutrient compositions on nocturnal glycemia in MDI-treated children and adolescents with T1D. Specifically, we compared carbohydrate-only (CHO), carbohydrate-plus-protein (CHO + PRO), carbohydrate-plus-fat (CHO + FAT), and carbohydrate-plus-fiber (CHO + FIB) bedtime snacks using CGM-derived nocturnal metrics, including TIR, TBR, time above range (TAR), GV, and overnight glucose profiles. We hypothesized that bedtime snacks differing in protein, fat, and fiber contents would yield distinct nocturnal CGM profiles, with the early postprandial period exhibiting the most evident differences. By clarifying which macronutrient combinations accompany the most stable early overnight glucose profiles, this study is intended to provide preliminary, practice-oriented evidence that may support more individualized bedtime snack counseling in pediatric diabetes care and inform the design of the adequately powered trials required before nutritional guidelines for MDI-treated youth can be updated.

## 2. Materials and Methods

### 2.1. Study Design and Participants

This prospective randomized crossover study evaluated the effects of bedtime snacks with different macronutrient compositions on nocturnal glycemic responses in MDI-treated children and adolescents with T1D. This study was conducted at the Pediatric Endocrinology and Diabetes Unit of Istanbul University–Cerrahpaşa, Cerrahpaşa Faculty of Medicine. The intervention was performed in the participants’ home setting under daily remote supervision by the study team through telephone contact.

Participants were recruited from children and adolescents with T1D receiving routine outpatient follow-up. The following were the inclusion criteria: aged 6–18 years, T1D duration beyond 1 year, remission period completion, treatment with MDI, completion of all three stages of carbohydrate-counting education, ability to perform capillary blood glucose monitoring at least four times daily, total daily insulin dose > 0.5 U/kg/day, and mean HbA1c < 9.6% during the preceding 3 months.

The following were the exclusion criteria: treatment with continuous subcutaneous insulin infusion (CSII), sensor-augmented pump therapy, or an automated insulin delivery (AID) system; incomplete carbohydrate-counting education, impaired awareness of hypoglycemia, intestinal malabsorption, delayed gastric emptying, celiac disease or any other dietary restriction-requiring condition, cystic fibrosis or other gastrointestinal disorders, diabetes-related chronic complications, physical or mental disability limiting adherence to study procedures, eating behavior disorders, and active infection.

A study form prepared by the investigator was employed for recording age, sex, age at diagnosis, diabetes duration, clinical presentation at diagnosis, family history of diabetes, current insulin regimen, daily basal and bolus insulin doses, total daily insulin dose, and insulin-to-carbohydrate ratios. Clinical data were verified using outpatient clinic records and electronic medical records. Body weight and height were obtained during routine clinical assessment, and body mass index was calculated as kg/m^2^. HbA1c and other biochemical parameters were obtained from laboratory results collected as part of routine clinical follow-up.

No a priori sample size calculation was performed. The number of participants was determined pragmatically, on the basis of the number of eligible children who could be supervised daily during a 12-night home-based protocol with blinded CGM and of the number of sensors available; the study was therefore designed and should be interpreted as an exploratory pilot (feasibility) trial intended to generate effect size estimates for subsequent adequately powered trials rather than to provide definitive between-snack comparisons.

### 2.2. Run-In Period and Standardization Procedures

Before the intervention, participants’ routine dietary records were reviewed for evaluating their usual evening meal patterns and individualized carbohydrate requirements. For study days, each participant was instructed to consume an evening meal containing the recommended amount of carbohydrates according to their usual meal plan and individualized insulin-to-carbohydrate ratio; meal boluses for main meals were calculated by the participants themselves, according to their usual carbohydrate-counting practice, and were administered with insulin lispro immediately before the meal. To preserve the home-based real-life nature of the intervention, a standardized evening meal was not provided. However, participants and caregivers were provided examples of appropriate evening meals for study days and were recommended to avoid fat- and/or protein-rich evening meals to reduce potential delayed effects on nocturnal glycemia.

Capillary blood glucose was measured by every participant immediately before the bedtime snack at 21:00, and the pre-snack value was documented on the daily record form together with the snack consumed. No a priori glucose threshold was applied for withholding the test snack or for excluding individual study nights; instead, participants managed pre-snack hypoglycemia or hyperglycemia according to their own standard, individualized diabetes management plan, and, in accordance with routine sick-day rules, checked ketones whenever capillary glucose exceeded 250 mg/dL. Bedtime (21:00) glucose values did not differ significantly across the four snack protocols (CHO, 124.0 ± 19.5 mg/dL; CHO + PRO, 130.6 ± 22.3 mg/dL; CHO + FAT, 134.0 ± 20.0 mg/dL; CHO + FIB, 119.8 ± 15.5 mg/dL; *p* = 0.230), and no study night was preceded by documented ketosis. Because each protocol was repeated on three consecutive nights and averaged at the participant level within a crossover design, occasional pre-snack hyperglycemia was expected to be distributed comparably across protocols; nevertheless, residual confounding by pre-snack glycemia and by the associated physiological insulin resistance cannot be fully excluded and is acknowledged in the limitations.

All participants used insulin glargine as the basal insulin and insulin lispro as the rapid-acting prandial insulin analog. Throughout this study, participants continued their usual basal–bolus MDI regimen; no changes were made to their standard diabetes treatment.

Participants were instructed to maintain their usual dietary pattern during the study period, except for the assigned bedtime snack. They were also instructed to weigh all foods consumed during the study period using a digital kitchen scale, compute carbohydrate content, and document their intake on dietary record forms. Additionally, their physical activities were recorded; however, they were asked to avoid vigorous physical activities during the intervention period.

### 2.3. Bedtime Snack Protocols

Four standardized whole-food bedtime snack protocols with different macronutrient compositions were prepared: Granny Smith apple as the carbohydrate-only snack (CHO); a mixture of SEK Protein Quark and SEK Strawberry Quark as the carbohydrate-plus-protein snack (CHO + PRO); Granny Smith apple with unsalted, unroasted nuts as the carbohydrate-plus-fat snack (CHO + FAT); and Wasa Fibre whole-grain rye crispbread as the carbohydrate-plus-fiber snack (CHO + FIB). The CHO snack consisted of 110 g of Granny Smith apple consumed with the peel. The CHO + PRO snack consisted of 140 g of SEK Protein Quark and 80 g of SEK Strawberry Quark. The CHO + FAT snack consisted of 90 g of Granny Smith apple and 48 g of mixed nuts comprising 36 g of almonds, 6 g of hazelnuts, and 6 g of walnuts. The CHO + FIB snack consisted of 37 g of Wasa Fibre whole-grain rye crispbread. All four snacks were matched for available carbohydrate (approximately 15–16 g) but, by design, were not matched for energy, because the aim was to compare whole-food snacks as consumed in everyday practice rather than isoenergetic formulations. All portions were weighed to the nearest gram using a digital kitchen scale supplied by the study team, and identical products were provided to every participant throughout the study. The energy and macronutrient composition of the test snacks, including the estimated fatty acid composition of the fat-containing snacks, is presented in Supplementary Table S1. The fat provided by the CHO + FAT snack was predominantly unsaturated and of nut origin, whereas the smaller amount of fat in the CHO + PRO snack was predominantly saturated and of dairy origin. This qualitative difference is considered in the Discussion.

The order of the four bedtime snack protocols was randomized for each participant using a simple lot-drawing procedure before the intervention period. Each participant consumed 12 test snacks on consecutive study days at 21:00 in the home setting. Each snack protocol was consumed for three consecutive nights. No washout period was included between snack protocols because the intervention comprised acute bedtime snacks and glycemic outcomes were assessed during the same overnight period following snack consumption. Participants were instructed to consume each bedtime snack within 10 min. The investigator verified snack consumption daily through telephone contact.

No additional bolus insulin was administered for the bedtime snacks. Following snack consumption, participants were asked not to consume any food or drink other than water unless treatment for hypoglycemia was necessitated. When hypoglycemia or hyperglycemia occurred at snack time or during the night, participants were permitted to intervene according to standard diabetes management recommendations. When capillary blood glucose levels were <70 mg/dL, participants were instructed to consume 0.3 g/kg of sucrose, recheck their blood glucose levels after 15 min, and repeat treatment when blood glucose levels remained <70 mg/dL.

### 2.4. CGM and Glycemic Outcomes

Glucose profiles were assessed using a blinded CGM system (Medtronic iPro™2; Medtronic MiniMed, Northridge, CA, USA). As the system provided retrospective glucose data, participants and caregivers were unable to view sensor glucose values in real time during the study. A trained diabetes nurse performed CGM device insertion. During the study period, each participant used three CGM sensors; data were uploaded to the Medtronic CareLink therapy management software (web-based platform) on sensor replacement days.

Throughout the study period, participants continued their routine capillary blood glucose monitoring. Capillary glucose measurements included pre- and post-meal values for breakfast, lunch, and dinner, as well as measurements at 23:00 and 03:00. Sensor calibration was performed four times daily using capillary blood glucose measurements according to the manufacturer’s recommendations. All participants used the same model of glucose meter (Ascensia Diabetes Care Holdings AG, Basel, Switzerland) for capillary glucose measurements.

Analyses included CGM data from the 12-h period between bedtime snack consumption at 21:00 and 09:00 the following morning, corresponding to 144 glucose readings per study day. The primary outcome was TIR (70–180 mg/dL) during the 21:00–09:00 period. To evaluate the time-dependent postprandial glycemic response, CGM-derived metrics were also analyzed for the 0–2-h, 2–4-h, 4–6-h, overall 0–6-h, and 6–12-h intervals following bedtime snack consumption.

Mean glucose, time in tight range (TITR; 70–140 mg/dL), TBR, TAR, coefficient of variation (%CV), peak glucose, nadir glucose, peak-to-nadir glucose amplitude, and incremental area under the glucose curve (iAUC) were secondary outcomes. Glucose values <70 mg/dL were categorized as TBR, with values <54 mg/dL classified as level 2 hypoglycemia. Glucose values >180 mg/dL were categorized as TAR, with values >250 mg/dL classified as level 2 hyperglycemia, according to the international consensus recommendations [13]. The %CV was calculated as the standard deviation (SD) of glucose values divided by the mean glucose value and multiplied by 100. iAUC was calculated using the trapezoidal rule relative to the glucose value at 21:00, immediately before bedtime snack consumption.

Furthermore, event-based hypoglycemia and hyperglycemia outcomes were assessed. Events were defined as consecutive CGM readings beyond the relevant threshold for at least 15 min, corresponding to at least three consecutive measurements. Episodes separated by <15 min were counted as a single event.

### 2.5. Statistical Analysis

Statistical analyses were performed using IBM SPSS Statistics (version 22.0; IBM Corp., Armonk, NY, USA). For each participant, the three nights recorded for each bedtime snack protocol were averaged into a single participant-level value to ensure that each participant contributed one observation per snack protocol. Therefore, comparisons across the four bedtime snack protocols were treated as within-subject repeated measures.

The Shapiro–Wilk test was employed for assessing the distribution of continuous variables, which were presented as means ± SDs or medians [interquartile ranges], as appropriate. Baseline characteristics were summarized using means ± SDs and medians (minimum–maximum) or medians (minimum–maximum) according to distribution. Categorical variables were expressed as numbers and percentages.

CGM-derived outcomes, including mean glucose, TIR, TITR, TBR, TAR, %CV, and glycemic stability metrics, were investigated for the overall overnight period and predefined postprandial intervals. Glycemic stability metrics encompassed peak glucose, nadir glucose, peak-to-nadir glucose amplitude, and iAUC.

Differences across the four bedtime snack protocols were tested using one-way repeated-measures analysis of variance and the Friedman test for normally and nonnormally distributed variables, respectively. When the omnibus test was statistically significant, pairwise comparisons were performed using paired-sample *t*-tests or Wilcoxon signed-rank tests, as appropriate, with Holm–Bonferroni correction for multiple comparisons. For significant pairwise differences in continuous outcomes, mean differences with 95% confidence intervals (CIs) were reported where applicable. Post hoc pairwise comparisons were performed only when the omnibus test was statistically significant, and their interpretation was restricted to comparisons that remained significant after Holm–Bonferroni adjustment; a significant omnibus test without a surviving pairwise difference was interpreted as an overall between-protocol difference that the present sample was too small to localize. The adjusted *p* values for all post hoc comparisons are reported in Supplementary Table S2.

Event-based hypoglycemia and hyperglycemia outcomes were summarized as total event counts and events per night. Using the Friedman test, within-participant event counts across the four snack protocols were compared. A two-sided *p*-value of <0.05 was considered statistically significant.

Because no a priori sample size calculation had been performed, a post hoc sensitivity analysis was conducted to establish the smallest effect that the available sample could detect. With 12 participants, four within-subject conditions, a two-sided α of 0.05, 80% power, an assumed average correlation of 0.5 among repeated measures, and assumed sphericity, the smallest detectable effect size for the omnibus repeated-measures comparison was f = 0.36 (partial η^2^ = 0.11); for unadjusted paired comparisons, the smallest detectable standardized mean difference was dz = 0.89, and the corresponding value for Holm–Bonferroni-adjusted comparisons is larger still. The study was therefore able to detect only large within-subject effects, and nonsignificant adjusted pairwise *p* values should be regarded as inconclusive (i.e., possibly reflecting Type II error) rather than as evidence of equivalence between protocols.

### 2.6. Ethical Approval

This study was conducted following the Declaration of Helsinki and was approved by the Clinical Research Ethics Committee of Acıbadem Mehmet Ali Aydınlar University on 26 June 2020 (approval number: 2020-13/5). The parents or legal guardians of all participants provided written informed consent; children and adolescents provided assent when appropriate. The study has since been retrospectively registered with the following details: Registry: ClinicalTrials.gov; Registration title: Effects of Bedtime Snack Composition on Overnight Glucose in Children and Adolescents With Type 1 Diabetes ClinicalTrials.gov Identifier: NCT07778147, Date first submitted: 13 August 2026, Date first posted: 21 August 2026.

## 3. Results

### 3.1. Participant Flow and Baseline Clinical Characteristics

**Figure 1:**
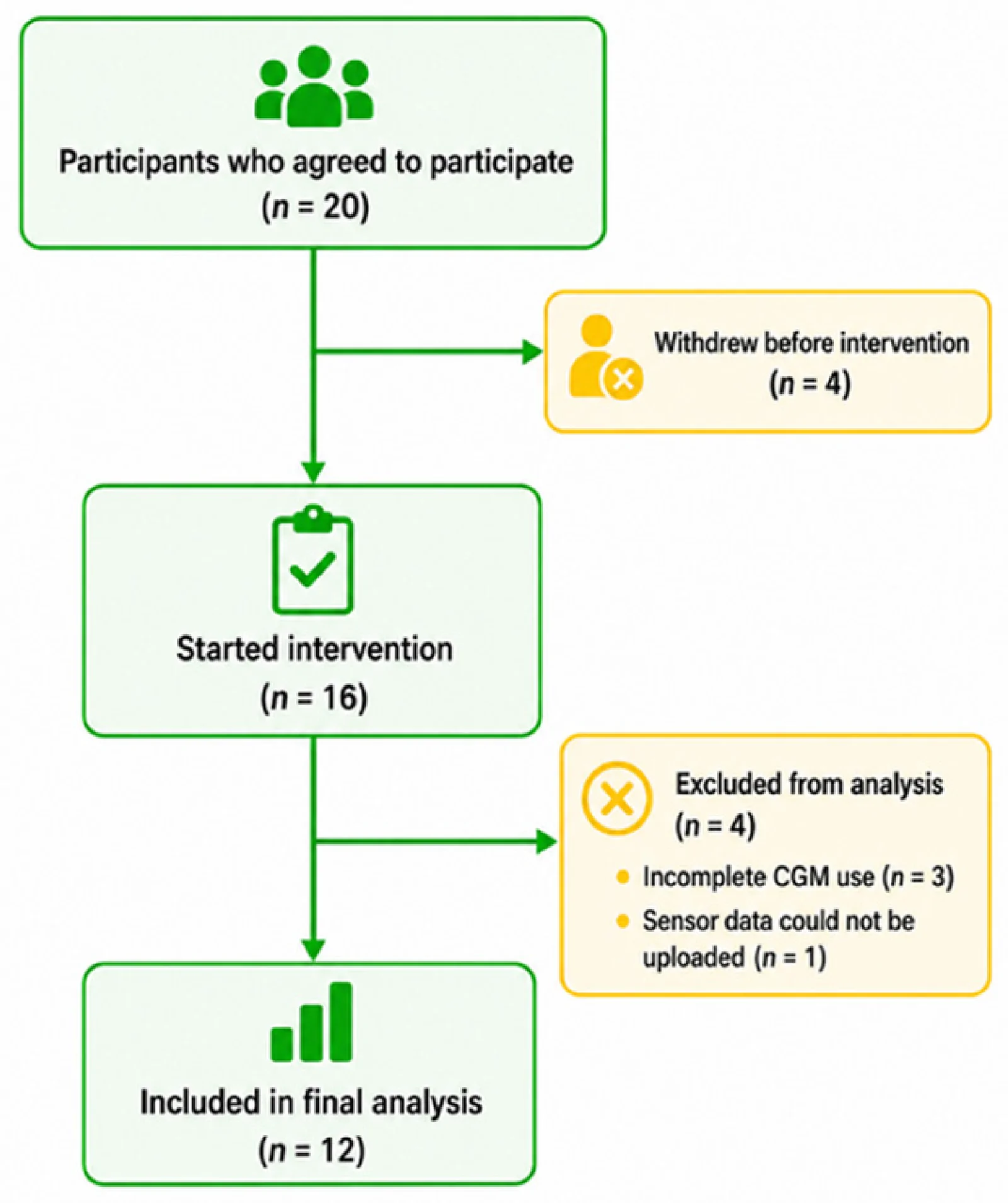
Flow diagram of participant enrollment, intervention completion, and inclusion in the final analysis.

Twenty participants initially agreed to participate in this study; however, four voluntarily withdrew before the intervention period commenced. Among the 16 participants who started the intervention, three were excluded owing to incomplete CGM use, and one was excluded because sensor glucose data could not be uploaded. Ultimately, 12 participants were included in the final analysis (Figure 1).

The final cohort comprised four females and eight males. The mean age, diabetes duration, and HbA1c were 12.8 ± 3.8 years, 5.1 ± 2.4 years, and 7.0% ± 0.6%, respectively. The baseline clinical characteristics of the participants are summarized in Table 1.

### 3.2. Overall Overnight Glycemic Control Between 21:00 and 09:00

Overnight TIR significantly differed across the four bedtime snack protocols during the 21:00–09:00 period (*p* = 0.014). The highest TIR was observed following the CHO + PRO snack protocol (85.8% ± 12.1%), whereas the lowest TIR was noted following the CHO + FAT snack protocol (70.9% ± 19.1%). TIR values following the CHO and CHO + FIB snack protocols were intermediate (82.3% ± 10.2% and 72.2% ± 17.3%, respectively).

However, following Holm–Bonferroni correction, none of the whole-night pairwise comparisons reached statistical significance. The lowest adjusted *p*-value was observed for CHO + PRO versus CHO + FAT (*p* = 0.079). Mean overnight glucose did not significantly differ across protocols (*p* = 0.516). Similarly, TITR, TBR, TAR, and the coefficient of variation (%CV, last row of Table 2) were comparable across protocols (Table 2).

### 3.3. Early Overnight Glycemic Responses According to Postprandial Time Intervals (First 6 h) and Across the Two Halves of the Night

Differences across the bedtime snack protocols were most pronounced during the first 6 h following snack consumption. These differences are presented sequentially for the three consecutive 2-h intervals that constitute the first 6 h (Table 3). During the first 2 h, neither mean glucose (*p* = 0.076) nor TIR (*p* = 0.423) differed significantly across protocols, whereas %CV did (*p* = 0.019), being lowest following CHO + PRO (7.7% ± 3.0%) and highest following CHO + FAT (12.4% ± 5.2%). Between 2 and 4 h, TIR differed across protocols (*p* = 0.008), amounting to 92.9% ± 6.7%, 94.0% ± 10.9%, 70.6% ± 31.3%, and 74.5% ± 25.0% following the CHO, CHO + PRO, CHO + FAT, and CHO + FIB protocols, respectively. A comparable pattern was observed between 4 and 6 h (TIR, *p* = 0.005), when TIR was again lowest following CHO + FAT (61.7% ± 29.3%). For each of these three intervals, no pairwise comparison remained statistically significant after Holm–Bonferroni adjustment (Supplementary Table S2).

When the overnight period was analyzed as two halves (Table 4), the between-protocol differences were confined to the first half of the night. During the 0–6-h interval, TIR was 89.0% ± 7.6%, 91.2% ± 7.6%, 71.4% ± 20.8%, and 77.9% ± 16.8% following the CHO, CHO + PRO, CHO + FAT, and CHO + FIB protocols, respectively (*p* = 0.001; ηp^2^ = 0.37); this was the only time-window metric presented in Table 4 for which a pairwise comparison remained statistically significant after Holm–Bonferroni adjustment, namely CHO + PRO versus CHO + FAT (adjusted *p* = 0.011). During the later 6–12-h interval, mean glucose (*p* = 0.729) and TIR (*p* = 0.260) were comparable across protocols, whereas %CV differed in the omnibus test (*p* = 0.046) without any pairwise difference surviving adjustment (Supplementary Table S2).

### 3.4. GV and Overnight Glycemic Stability

Whole-night GV was numerically the lowest following the CHO + PRO snack protocol. Overnight CV was 18.0% ± 4.6% following CHO + PRO, compared with 23.5% ± 8.3%, 24.3% ± 8.7%, and 22.7% ± 8.4% following the CHO, CHO + FAT, and CHO + FIB protocols, respectively; however, this difference did not reach statistical significance (*p* = 0.059; %CV values for the whole night are reported in the last row of Table 2).

Peak-to-nadir glucose amplitude significantly differed across the bedtime snack protocols (*p* = 0.025). The lowest amplitude was observed following CHO + PRO (88.1 ± 24.9 mg/dL), whereas the highest amplitude was noted following CHO + FAT (123.9 ± 36.5 mg/dL). Amplitude values following CHO and CHO + FIB were intermediate (110.8 ± 31.6 and 106.0 ± 40.8 mg/dL, respectively).

In Holm–Bonferroni-adjusted pairwise comparisons, the only significant difference was between the CHO + PRO and CHO + FAT protocols (*p* = 0.041; mean difference, −35.9 mg/dL; 95% CI, −59.7 to −12.1). Peak glucose, nadir glucose, and iAUC values did not significantly differ across the snack protocols; peak-to-nadir amplitude is reported in the third row of Table 5.

### 3.5. Clinical Safety

Event-based hypoglycemia and hyperglycemia outcomes did not significantly differ across the bedtime snack protocols. The rates of hypoglycemic events <70 mg/dL were 0.33, 0.11, 0.39, and 0.47 events/night following the CHO, CHO + PRO, CHO + FAT, and CHO + FIB protocols, respectively (*p* = 0.185). No level 2 hypoglycemic events (<54 mg/dL) were noted following the CHO + PRO protocol, whereas one event was recorded following CHO, and five events each were recorded following the CHO + FAT and CHO + FIB protocols (*p* = 0.154). Hyperglycemic events (>180 mg/dL) were comparable across the protocols, with 25, 25, 36, and 22 events following the CHO, CHO + PRO, CHO + FAT, and CHO + FIB protocols, respectively (*p* = 0.179). Level 2 hyperglycemic events (>250 mg/dL) were not observed following the CHO + PRO protocol, whereas two, six, and eight events, respectively, were documented following the CHO, CHO + FAT, and CHO + FIB protocols, respectively (*p* = 0.069) (Table 6).

### 3.6. Nocturnal Glucose Trajectories and Individual Variability

**Figure 2:**
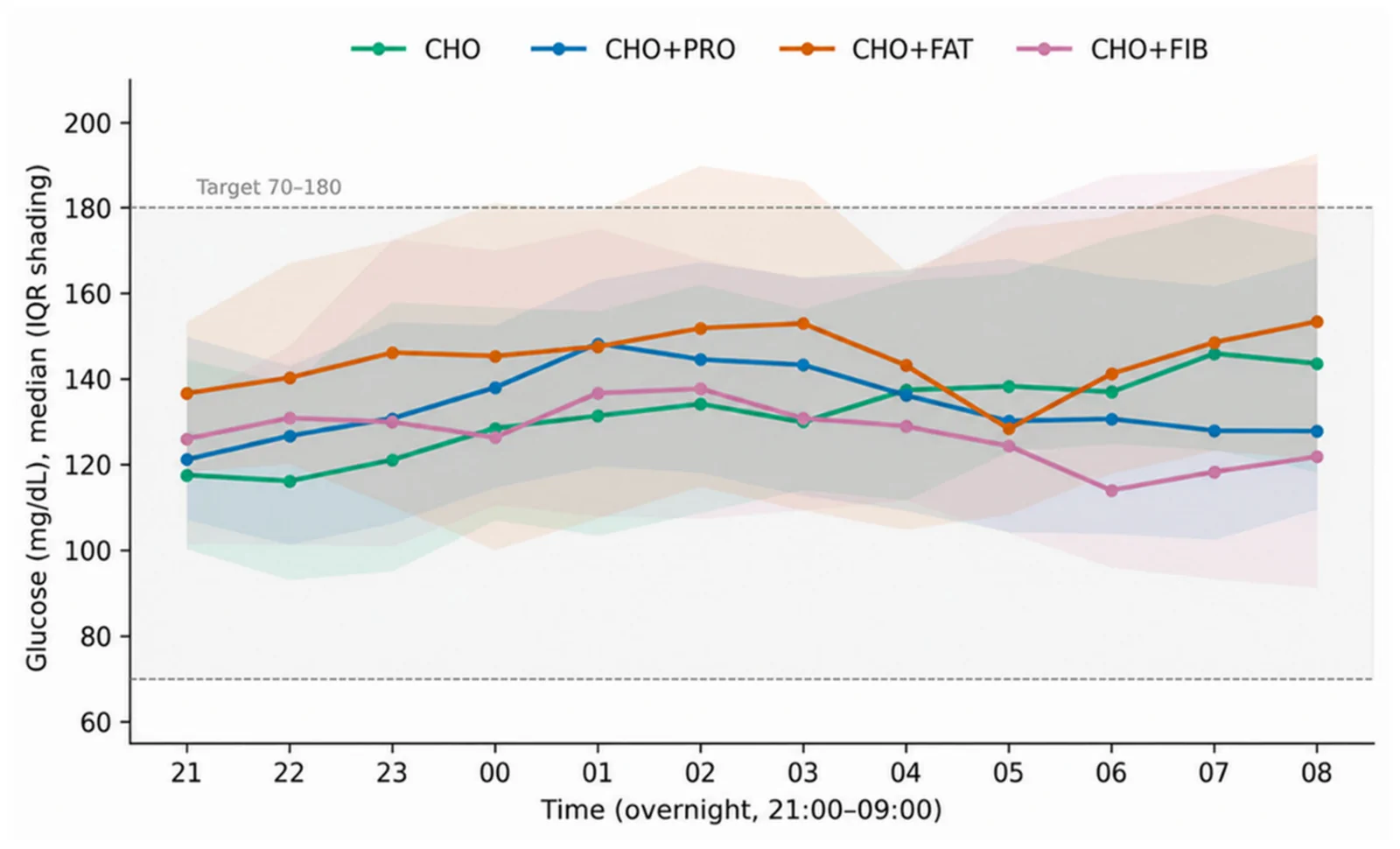
Mean nocturnal glucose trajectories between 21:00 and 09:00. Lines indicate hourly mean glucose values for each bedtime snack protocol, and shaded areas represent the night-level interquartile range (*n* = 36 nights per protocol). The gray band represents the target glucose range of 70–180 mg/dL.

**Figure 3:**
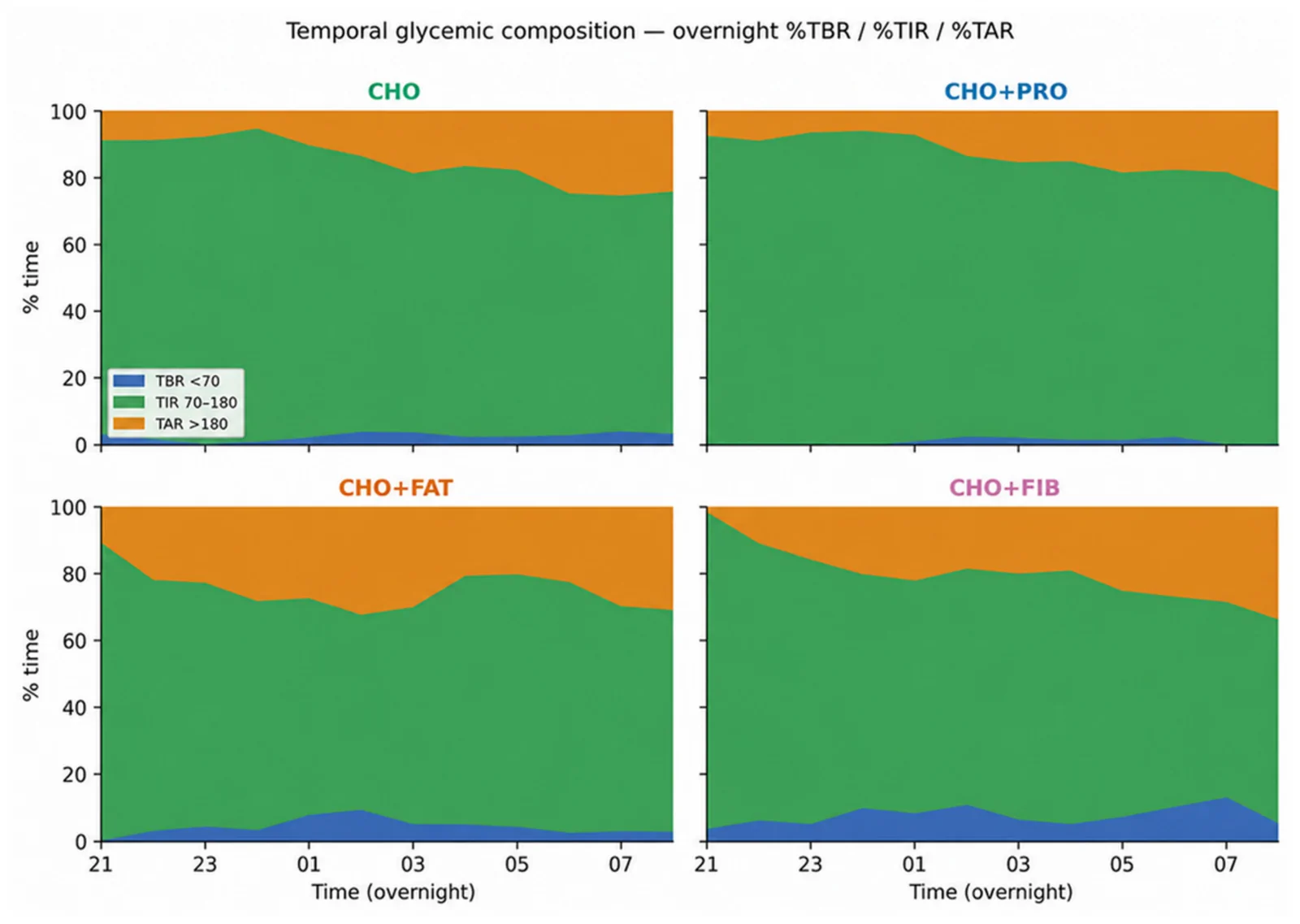
Temporal glycemic composition during the overnight period. The hourly distribution of time below range (%TBR, <70 mg/dL), time in range (%TIR, 70–180 mg/dL), and time above range (%TAR, >180 mg/dL) is displayed for each bedtime snack protocol.

**Figure 4:**
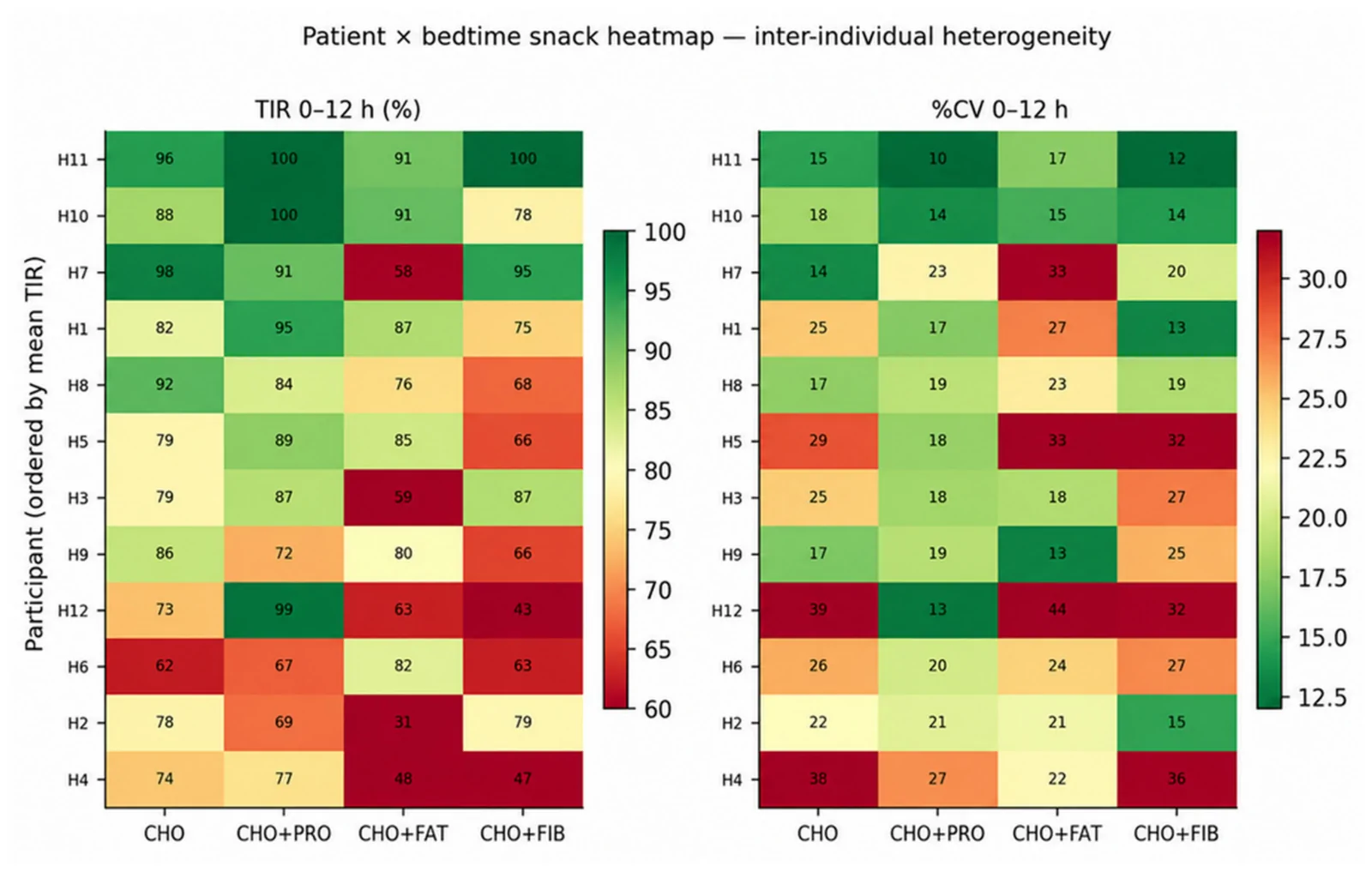
Patient-by-snack heatmaps during the 0–12-h overnight period. The left and right panels indicate TIR (%) and CV (%), respectively. Each row represents an individual participant, and each column represents a bedtime snack protocol. Participants are arranged in descending order according to mean TIR.

Across all bedtime snack protocols, hourly glucose trajectories remained largely within the target range. Mean glucose values appeared relatively higher during parts of the later overnight period following the CHO + FAT protocol, whereas the CHO + PRO protocol exhibited a narrower glycemic profile over time (Figure 2). The hourly distribution of TBR, TIR, and TAR across snack protocols is depicted in Figure 3. Patient-by-snack heatmaps revealed substantial interindividual variability in both TIR and CV responses to the bedtime snack protocols (Figure 4).

## 4. Discussion

In this randomized crossover study evaluating bedtime snacks with different macronutrient compositions in MDI-treated children and adolescents with T1D, nocturnal glycemic responses varied according to snack composition. The CHO + PRO protocol was associated with the highest numerical whole-night TIR and the lowest GV; however, adjusted whole-night pairwise comparisons were not statistically significant. The clearest between-protocol difference was noted during the first 6 h following snack consumption, when CHO + PRO was associated with a significantly higher TIR than CHO + FAT. Moreover, CHO + PRO was associated with a significantly lower peak-to-nadir glucose amplitude than CHO + FAT, suggesting a more stable early nocturnal glucose profile. By contrast, mean glucose levels and event-based hypoglycemia and hyperglycemia outcomes did not significantly differ across protocols. Therefore, these findings should be interpreted as preliminary evidence that bedtime snack composition may impact early nocturnal glycemic stability, rather than as definitive evidence of superiority of any single bedtime snack strategy.

The clinical relevance of the observed TIR difference requires cautious interpretation. International consensus recommendations highlight TIR as a clinically meaningful outcome, and an absolute difference of approximately 5% in TIR has been proposed as a useful threshold for clinical interpretation [13]. In this study, the early night TIR difference between CHO + PRO and CHO + FAT exceeded this threshold. However, whole-night pairwise differences in TIR did not remain statistically significant following adjustment for multiple comparisons, and the sample size was small. Therefore, although the magnitude of the early night difference is potentially clinically relevant, it should be considered hypothesis-generating and warrants validation in larger studies.

A clear distinction should also be drawn between omnibus and pairwise statistical significance. Significant omnibus tests were obtained for whole-night TIR, for TIR during the 2–4-h and 4–6-h intervals, and for %CV during the 0–2-h and 6–12-h intervals; none of these differences, however, remained significant at the pairwise level after Holm–Bonferroni correction. Only two adjusted pairwise comparisons remained significant, and both contrasted CHO + PRO with CHO + FAT (TIR during 0–6 h and peak-to-nadir glucose amplitude). The present data therefore indicate an overall difference among the four protocols that the available sample was too small to localize for most outcomes, and no claim of superiority is advanced for any protocol beyond these two comparisons. In particular, the numerically higher TIR and lower variability observed after CHO + PRO must not be read as evidence that a protein-containing bedtime snack protects against nocturnal hypoglycemia.

The necessity and optimal composition of bedtime snacks in T1D remain debated, and the available evidence remains scarce. Desjardins et al. concluded that existing evidence was inadequate to determine the benefit and ideal composition of bedtime nutritional strategies, particularly in individuals managed with modern insulin analogs [7]. Similarly, ISPAD nutritional guidance supports nutritional management tailored according to clinical context, treatment regimen, glycemic patterns, and patient needs, rather than a universal bedtime snack prescription [9]. Our findings partly address this evidence gap by comparing standardized whole-food bedtime snacks with different macronutrient profiles in a pediatric MDI population, although they do not resolve it.

Our findings should also be distinguished from studies evaluating the relevance of bedtime snacks. Gökçe et al. reported that bedtime snacks with comparable macronutrient content, including milk, yogurt, or kefir, reduced TIR during the first 6 h and did not decrease hypoglycemia frequency in MDI-treated young children with T1D [14]. Their study directly compared bedtime snack conditions with a no-snack control condition, whereas our study compared various bedtime snack compositions when a snack was provided. Therefore, the two studies address related but distinct clinical questions. Furthermore, age range, snack composition, study design, and baseline glycemic profile differences may have contributed to the inconsistent findings. Overall, these data support a cautious and individualized approach rather than routine bedtime snack recommendations for all children with T1D.

Evidence on protein-containing bedtime snacks remains lacking. Our finding that CHO + PRO was associated with higher early night TIR and lower glucose amplitude than CHO + FAT broadly aligns with previous evidence suggesting that protein-containing bedtime snacks can affect nocturnal glucose profiles in T1D [15]. In this study, no level 2 hypoglycemic events (<54 mg/dL) and no level 2 hyperglycemic events (>250 mg/dL) were noted following CHO + PRO. However, this observation should be interpreted as a reassuring descriptive finding rather than as evidence of protection, because event counts were low and between-protocol differences were not statistically significant.

The temporal pattern observed following CHO + PRO may be biologically plausible; however, the underlying mechanisms cannot be identified on the basis of the present data. Protein-containing evening meals may impact nocturnal glucose regulation through delayed substrate availability, gluconeogenesis, and hormonal responses, including glucagon secretion [16]. In this study, the more favorable glucose profile associated with CHO + PRO was most evident during the first half of the night, whereas differences in TIR were less apparent during the later 6–12-h interval. This pattern potentially reflects the interaction among snack composition, basal insulin action, and delayed nutrient metabolism. However, insulin, glucagon, free fatty acids, gastric emptying, and other metabolic markers were not assessed. Therefore, mechanistic explanations remain speculative and require investigation in future studies. The mechanisms outlined above should accordingly be regarded as potential explanations derived from the existing literature and not as processes demonstrated by the present study; our data can show only that glucose profiles potentially differ according to snack composition, not why they do so.

By contrast, CHO + FAT demonstrated the least favorable glycemic profile across the tested protocols, with the lowest TIR and the highest peak-to-nadir glucose amplitude. This finding does not imply that all fat-containing bedtime snacks are unfavorable; rather, the specific CHO + FAT composition investigated in this study did not appear to offer a glycemic advantage when no additional bolus insulin was administered for the snack. Previous pediatric evidence comparing low-fat and high-fat bedtime snacks in children and adolescents with T1D did not observe significant differences in nocturnal hypoglycemia or hyperglycemia [10]. Conversely, observational data from adults with T1D have associated postdinner dietary intake, including fat intake, with nocturnal glycemic risk [17]. Collectively, the available evidence suggests that meal context, insulin dosing, timing, and individual metabolic responses can influence the effects of fat-containing bedtime snacks.

A further consideration when interpreting the CHO + FAT results is that the four snacks were matched for available carbohydrate but deliberately not for energy. The CHO + FAT snack provided 350 kcal, compared with 64 kcal for CHO, 122 kcal for CHO + FIB, and 190 kcal for CHO + PRO, corresponding to an approximately 5.5-fold range in energy load. Total energy, fat quantity, and food matrix are therefore confounded in this comparison. Because gastric emptying is strongly influenced by the caloric density of the ingested meal, whereas the nature of the calories appears to play a comparatively smaller role, the substantially greater energy load of the CHO + FAT snack may have prolonged gastric retention and delayed the appearance of exogenous glucose [18]. This possibility cannot be resolved with the present design, and the CHO + FAT arm is best interpreted as an “energy-dense, nut-based bedtime snack” rather than as “fat” in isolation. Future studies using carbohydrate-matched, isoenergetic snack protocols are required to disentangle the effects of total energy content from those of macronutrient composition and food matrix.

The composition of the test foods also warrants comment. The snacks were selected to reflect items that families actually use at bedtime rather than to constitute a factorial manipulation of single macronutrients; consequently, for each macronutrient of interest, one snack contained a high amount and one contained essentially none, while the remaining two provided intermediate amounts (Supplementary Table S1). Between-protocol contrasts thus reflect the combined effects of whole foods, including their matrix, texture, and rate of digestion, rather than the isolated effect of protein, fat, or fiber. The quality of the fat also differed between protocols: the fat in the CHO + FAT snack was predominantly unsaturated and of nut origin, whereas the smaller quantity of fat in the CHO + PRO snack was largely saturated and of dairy origin. In people with T1D, a meal rich in monounsaturated fat has been shown to modify gastric emptying and elicit a greater GLP-1 response than a meal rich in saturated fat [19]. Nevertheless, the glucose profile observed after CHO + FAT in the present study cannot be attributed to fatty acid class alone, because the nut matrix, fiber and polyphenol content, and substantially greater energy load may also have contributed.

GV findings aligned with the possibility that CHO + PRO generated a more stable nocturnal glucose profile. Although whole-night CV was lowest following CHO + PRO, the difference was not statistically significant. By contrast, peak-to-nadir glucose amplitude significantly differed across snack protocols; the adjusted pairwise comparison between CHO + PRO and CHO + FAT remained significant. This finding is crucial because mean overnight glucose levels were comparable across snack protocols. Therefore, mean glucose alone may insufficiently capture differences in the magnitude of nocturnal glucose excursions. These findings support the value of CGM-derived variability and stability metrics when evaluating nutritional interventions in T1D [13].

The nocturnal glucose trajectory and temporal glycemic composition figures offer descriptive support for these findings. Across snack protocols, mean hourly glucose values generally remained within the target range, whereas CHO + PRO exhibited a narrower glycemic profile over time, and CHO + FAT appeared to be associated with relatively higher glucose values during parts of the night. Furthermore, patient-by-snack heatmaps revealed substantial interindividual variability in both TIR and CV responses. Some participants appeared to respond more favorably to one snack composition, whereas others exhibited less consistent patterns across protocols. This heterogeneity is clinically plausible because multiple factors, including basal insulin pharmacodynamics, insulin sensitivity, pubertal status, evening meal composition, prior physical activity, and bedtime glucose level, can influence nocturnal glycemia in children and adolescents with T1D. However, this study was not designed or powered to identify predictors of individual responses. Therefore, these findings should be considered hypothesis-generating for future studies evaluating individualized bedtime snack strategies.

This study had several strengths. The randomized crossover design enabled each participant to serve as their own control, reducing between-person variability. The use of blinded CGM facilitated a comprehensive assessment of overnight glucose profiles while minimizing behavioral changes that might develop when participants or caregivers could view sensor glucose values in real time. Moreover, the use of standardized whole-food bedtime snacks increased the practical relevance of the findings for routine pediatric diabetes management. Daily remote supervision, snack verification, and dietary recording also supported adherence to the study protocol.

Several limitations should be acknowledged. First, this was a single-center study with a small final sample size, which limited statistical power, the precision of effect estimates, and the ability to detect smaller between-protocol differences, particularly following correction for multiple comparisons. A post hoc sensitivity analysis indicated that only large within-subject effects (f ≥ 0.36; dz ≥ 0.89) could have been detected, and nonsignificant pairwise comparisons should therefore not be interpreted as evidence of no difference. Because no a priori power calculation was performed, the study is best regarded as an exploratory pilot trial. Although the effect size observed for 0–6-h TIR suggests the potential relevance of the early night difference, this finding warrants validation in sufficiently powered studies. Second, the absence of a no-snack control arm precluded conclusions about whether bedtime snacking is necessary or beneficial; the present findings only compared glycemic responses to different bedtime snack compositions when a snack is consumed. Third, although participants were instructed to consume the recommended carbohydrate amount at the evening meal and avoid high-fat and high-protein meals during the study period, evening meals were not fully standardized under controlled laboratory conditions. Therefore, residual variation in evening meal composition potentially impacted nocturnal glycemic responses. Fourth, all participants were treated with glargine/lispro-based MDI and demonstrated relatively good baseline glycemic control. Therefore, the findings may not be directly generalizable to children and adolescents using insulin pumps, sensor-augmented pump therapy, or AID systems, or to those with poorer metabolic control and a higher risk of nocturnal dysglycemia. Fifth, no additional bolus insulin was administered for the bedtime snacks; therefore, the results may not apply to strategies in which bedtime snacks are covered with insulin. Metabolic markers, including insulin, glucagon, free fatty acids, and gastric emptying, were also not assessed, preventing mechanistic interpretation of the observed glycemic patterns. Sixth, the four snacks were matched for carbohydrate but not for energy, so the effects attributed to fat may partly reflect the greater energy load of the CHO + FAT arm; total energy intake and macronutrient composition cannot be disentangled in this design. Seventh, although pre-snack capillary glucose was recorded and did not differ across protocols, bedtime glycemia and the antecedent evening meal were not controlled experimentally, and residual confounding by pre-snack hyperglycemia and the associated physiological insulin resistance cannot be excluded. Finally, the sequence of snack protocols was randomized by simple lot drawing without allocation concealment, and, because the protocols were run on consecutive nights without a washout period, carry-over effects between adjacent protocols cannot be formally excluded.

## 5. Conclusions

Among children and adolescents with T1D treated with glargine/lispro-based MDI, bedtime snack composition was associated with differences in early nocturnal glycemic stability. The CHO + PRO protocol was associated with higher TIR during the first 6 h following snack consumption and lower peak-to-nadir glucose amplitude than the CHO + FAT protocol. However, whole-night pairwise TIR differences did not remain statistically significant following adjustment for multiple comparisons, and event-based hypoglycemia and hyperglycemia outcomes did not significantly vary across protocols.

Notably, as this study did not include a no-snack control condition, the findings do not establish the necessity or benefit of routine bedtime snacking. Rather, they suggest that macronutrient composition may potentially influence early overnight glycemic stability when a bedtime snack is clinically indicated. These results should be interpreted with caution: only two pairwise comparisons remained statistically significant after correction for multiple testing, the snacks were not matched for energy, and the sample was small. Accordingly, the findings must not be taken to indicate that a protein-containing bedtime snack protects against nocturnal hypoglycemia, and they do not support the routine substitution of one snack composition for another in clinical practice at this stage. Larger adequately powered studies, including no-snack control conditions, broader glycemic risk profiles, different insulin delivery modalities, and mechanistic assessments, are required to validate these preliminary findings.

## Figures and Tables

**Table 1:** Baseline clinical characteristics of the participants.

Variable	Total Cohort (*n* = 12)
Age, years	12.8 ± 3.8; 13.5 (6–17)
BMI SDS	−0.50 ± 0.66; −0.37 (−1.43 to 0.72)
Age at diagnosis, years	7.7 ± 3.8; 9.5 (2–13)
Diabetes duration, years	5.1 ± 2.4; 5.0 (2–9)
HbA1c, %	7.01 ± 0.6; 7.01 (5.80–7.90)
Total daily insulin dose, U/kg/day	0.94 ± 0.19; 0.94 (0.60–1.23)
Bolus insulin, % of the total daily dose	53.4 (40.0–66.7)
Basal insulin, % of the total daily dose	47.6 (33.3–60.0)
Insulin sensitivity factor, mg/dL per unit	50.0 (35.0–90.0)

Data are presented as means ± SDs and medians (minimum–maximum) or medians (minimum–maximum) as appropriate. BMI SDS, body mass index standard deviation score; HbA1c, glycated hemoglobin A1c.

**Table 2:** CGM-derived glycemic outcomes during the overall overnight period (21:00–09:00).

Metric	CHO	CHO + PRO	CHO + FAT	CHO + FIB	*p*
Mean glucose, mg/dL	137.6 ± 13.3	136.7 ± 15.9	146.9 ± 27.4	137.3 ± 27.5	0.516
TIR 70–180 mg/dL, %	82.3 ± 10.2	85.8 ± 12.1	70.9 ± 19.1	72.2 ± 17.3	0.014 *
TITR 70–140 mg/dL, %	55.2 ± 11.9	54.7 ± 20.0	44.1 ± 22.4	53.9 ± 20.6	0.275
TBR < 70 mg/dL, %	0.6 [0.0–3.2]	0.0 [0.0–0.2]	0.3 [0.0–6.4]	3.6 [0.0–9.3]	0.148
TBR < 54 mg/dL, %	0.0 [0.0–0.0]	0.0 [0.0–0.0]	0.0 [0.0–0.5]	0.0 [0.0–0.8]	0.154
TAR > 180 mg/dL, %	14.8 ± 9.1	12.9 ± 11.5	24.4 ± 19.6	19.9 ± 16.6	0.230
TAR > 250 mg/dL, %	0.0 [0.0–0.0]	0.0 [0.0–0.0]	0.0 [0.0–1.1]	0.0 [0.0–6.1]	0.439
CV, %	23.5 ± 8.3	18.0 ± 4.6	24.3 ± 8.7	22.7 ± 8.4	0.059

Data are presented as means ± SDs or medians [interquartile ranges] as appropriate. Comparisons are performed using repeated-measures ANOVA or the Friedman test as appropriate. ANOVA, analysis of variance; CGM, continuous glucose monitoring; TIR, time in range; TITR, time in tight range; TBR, time below range; TAR, time above range; CV, coefficient of variation. * *p* < 0.05. Post hoc pairwise comparisons are adjusted using the Holm–Bonferroni method and are provided in Supplementary Table S2.

**Table 3:** CGM-derived glycemic outcomes during the first 6 h after the bedtime snack, according to 2-h postprandial intervals.

Metric	CHO	CHO + PRO	CHO + FAT	CHO + FIB	*p*
0–2 h					
Mean glucose, mg/dL	122.4 ± 14.2	129.3 ± 19.2	141.5 ± 24.2	125.1 ± 21.7	0.076
TIR 70–180 mg/dL, %	95.8 [86.8–100.0]	98.6 [85.4–100.0]	84.7 [74.3–96.9]	93.8 [81.3–100.0]	0.423
TITR 70–140 mg/dL, %	69.4 ± 25.8	67.6 ± 22.5	51.9 ± 26.1	66.3 ± 24.6	0.176
TBR < 70 mg/dL, %	0.0 [0.0–3.8]	0.0 [0.0–0.0]	0.0 [0.0–1.0]	0.0 [0.0–8.3]	0.475
TBR < 54 mg/dL, %	0.0 [0.0–0.0]	0.0 [0.0–0.0]	0.0 [0.0–0.0]	0.0 [0.0–0.0]	0.392
TAR > 180 mg/dL, %	8.4 ± 12.9	7.9 ± 11.5	16.0 ± 19.6	5.8 ± 12.1	0.223
TAR > 250 mg/dL, %	0.0 [0.0–0.0]	0.0 [0.0–0.0]	0.0 [0.0–0.0]	0.0 [0.0–0.0]	0.572
CV, %	10.3 ± 2.7	7.7 ± 3.0	12.4 ± 5.2	10.2 ± 3.7	0.019 *
2–4 h					
Mean glucose, mg/dL	129.1 ± 18.7	133.5 ± 16.6	147.4 ± 39.6	140.4 ± 39.6	0.456
TIR 70–180 mg/dL, %	92.9 ± 6.7	94.0 ± 10.9	70.6 ± 31.3	74.5 ± 25.0	0.008 *
TITR 70–140 mg/dL, %	61.0 ± 22.8	60.5 ± 23.1	45.3 ± 32.9	53.1 ± 23.2	0.279
TBR < 70 mg/dL, %	0.0 [0.0–0.0]	0.0 [0.0–0.0]	0.0 [0.0–0.7]	0.0 [0.0–16.7]	0.148
TBR < 54 mg/dL, %	0.0 [0.0–0.0]	0.0 [0.0–0.0]	0.0 [0.0–0.0]	0.0 [0.0–0.0]	0.145
TAR > 180 mg/dL, %	4.2 [0.0–10.1]	0.0 [0.0–4.9]	10.4 [0.0–37.8]	7.6 [0.0–20.8]	0.268
TAR > 250 mg/dL, %	0.0 [0.0–0.0]	0.0 [0.0–0.0]	0.0 [0.0–0.0]	0.0 [0.0–0.0]	0.261
CV, %	10.3 ± 3.2	6.6 ± 2.6	10.7 ± 6.3	9.8 ± 4.3	0.055
4–6 h					
Mean glucose, mg/dL	134.1 ± 23.2	143.4 ± 18.5	148.8 ± 40.4	141.5 ± 35.5	0.689
TIR 70–180 mg/dL, %	85.1 ± 14.6	87.8 ± 11.4	61.7 ± 29.3	70.1 ± 20.3	0.005 *
TITR 70–140 mg/dL, %	55.8 ± 28.5	41.8 ± 29.9	36.9 ± 38.6	43.3 ± 27.8	0.512
TBR < 70 mg/dL, %	0.0 [0.0–1.0]	0.0 [0.0–0.0]	0.0 [0.0–11.8]	0.0 [0.0–10.1]	0.461
TBR < 54 mg/dL, %	0.0 [0.0–0.0]	0.0 [0.0–0.0]	0.0 [0.0–0.0]	0.0 [0.0–0.0]	0.194
TAR > 180 mg/dL, %	11.6 ± 14.4	10.1 ± 11.1	29.5 ± 27.1	20.0 ± 17.7	0.108
TAR > 250 mg/dL, %	0.0 [0.0–0.0]	0.0 [0.0–0.0]	0.0 [0.0–0.7]	0.0 [0.0–0.7]	0.112
CV, %	7.7 ± 2.2	5.8 ± 1.8	7.7 ± 4.3	6.7 ± 3.1	0.295

Data are presented as means ± SDs or medians [interquartile ranges] as appropriate. Comparisons are performed using repeated-measures ANOVA or the Friedman test as appropriate. TIR, time in range; TITR, time in tight range; TBR, time below range; TAR, time above range; CV, coefficient of variation. * *p* < 0.05. Post hoc pairwise comparisons are adjusted using the Holm–Bonferroni method and are provided in Supplementary Table S2.

**Table 4:** CGM-derived glycemic outcomes during the two halves of the overnight period (0–6 h and 6–12 h after the bedtime snack).

Metric	CHO	CHO + PRO	CHO + FAT	CHO + FIB	*p*
0–6 h					
Mean glucose, mg/dL	128.5 ± 14.3	135.4 ± 14.6	145.9 ± 31.1	135.7 ± 30.7	0.345
TIR 70–180 mg/dL, %	89.0 ± 7.6	91.2 ± 7.6	71.4 ± 20.8	77.9 ± 16.8	0.001 **
TITR 70–140 mg/dL, %	62.1 ± 17.3	56.6 ± 21.2	44.7 ± 26.1	54.2 ± 21.1	0.214
TBR < 70 mg/dL, %	0.0 [0.0–3.9]	0.0 [0.0–0.0]	0.0 [0.0–4.2]	0.0 [0.0–11.2]	0.347
TBR < 54 mg/dL, %	0.0 [0.0–0.0]	0.0 [0.0–0.0]	0.0 [0.0–0.0]	0.0 [0.0–0.0]	0.194
TAR > 180 mg/dL, %	8.8 ± 7.4	8.0 ± 6.9	23.5 ± 20.9	14.5 ± 17.1	0.076
TAR > 250 mg/dL, %	0.0 [0.0–0.0]	0.0 [0.0–0.0]	0.0 [0.0–1.2]	0.0 [0.0–0.2]	0.063
CV, %	18.7 ± 3.5	13.8 ± 5.2	20.2 ± 7.9	18.7 ± 7.4	0.085
6–12 h					
Mean glucose, mg/dL	146.6 ± 28.6	138.1 ± 22.5	147.9 ± 26.3	139.0 ± 36.8	0.729
TIR 70–180 mg/dL, %	75.7 ± 16.6	80.4 ± 19.5	70.4 ± 21.7	66.4 ± 25.3	0.260
TITR 70–140 mg/dL, %	48.3 ± 20.8	52.8 ± 27.1	43.4 ± 25.4	53.5 ± 29.5	0.591
TBR < 70 mg/dL, %	0.0 [0.0–2.3]	0.0 [0.0–0.3]	0.0 [0.0–7.1]	0.0 [0.0–9.1]	0.576
TBR < 54 mg/dL, %	0.0 [0.0–0.0]	0.0 [0.0–0.0]	0.0 [0.0–1.0]	0.0 [0.0–0.0]	0.252
TAR > 180 mg/dL, %	20.8 ± 17.0	17.8 ± 19.0	25.3 ± 21.5	25.2 ± 25.1	0.649
TAR > 250 mg/dL, %	0.0 [0.0–0.0]	0.0 [0.0–0.0]	0.0 [0.0–0.6]	0.0 [0.0–2.2]	0.460
CV, %	13.5 ± 6.2	10.2 ± 4.2	16.7 ± 8.6	13.5 ± 6.5	0.046 *

Data are presented as means ± SDs or medians [interquartile ranges] as appropriate. Comparisons are performed using repeated-measures ANOVA or the Friedman test as appropriate. TIR, time in range; TITR, time in tight range; TBR, time below range; TAR, time above range; CV, coefficient of variation. * *p* < 0.05; ** *p* < 0.005. Post hoc pairwise comparisons are adjusted using the Holm–Bonferroni method and are provided in Supplementary Table S2.

**Table 5:** Glycemic stability and kinetic characteristics during the overall overnight period.

Metric	CHO	CHO + PRO	CHO + FAT	CHO + FIB	*p*
Peak glucose, mg/dL	194.5 ± 26.4	183.9 ± 26.6	215.9 ± 38.5	192.2 ± 46.5	0.122
Nadir glucose, mg/dL	83.7 ± 14.0	95.8 ± 12.1	92.0 ± 25.8	86.2 ± 18.4	0.350
Amplitude, peak–nadir, mg/dL	110.8 ± 31.6	88.1 ± 24.9	123.9 ± 36.5	106.0 ± 40.8	0.025 *
Total iAUC, mg/dL·h	161.3 ± 246.3	73.9 ± 198.3	153.9 ± 364.3	209.6 ± 291.2	0.642
Early iAUC, 0–6 h, mg/dL·h	26.4 ± 126.6	28.3 ± 95.5	70.7 ± 196.8	94.6 ± 162.0	0.581
Late iAUC, 6–12 h, mg/dL·h	133.9 ± 193.2	44.5 ± 133.5	82.0 ± 178.8	113.5 ± 208.2	0.520

Data are presented as means ± SDs. Comparisons are performed using repeated-measures ANOVA. iAUC, incremental area under the curve; CI, confidence interval. * *p* < 0.05. Post hoc pairwise comparisons are adjusted using the Holm–Bonferroni method. For amplitude, the adjusted comparison between CHO + PRO and CHO + FAT is significant (*p* = 0.041; mean difference, −35.9 mg/dL; 95% CI, −59.7 to −12.1).

**Table 6:** Event-based hypoglycemia and hyperglycemia outcomes during the overall overnight period.

Event Count, *n* (per Night), Total 36 Nights	CHO	CHO + PRO	CHO + FAT	CHO + FIB	*p*
Hypoglycemic events < 70 mg/dL	12 (0.33)	4 (0.11)	14 (0.39)	17 (0.47)	0.185
Level 2 hypoglycemia < 54 mg/dL	1 (0.03)	0 (0.00)	5 (0.14)	5 (0.14)	0.154
Hyperglycemic events > 180 mg/dL	25 (0.69)	25 (0.69)	36 (1.00)	22 (0.61)	0.179
Level 2 hyperglycemia > 250 mg/dL	2 (0.06)	0 (0.00)	6 (0.17)	8 (0.22)	0.069

Event definition according to the Battelino et al. 2019 [13] consensus: consecutive readings beyond the threshold for ≥15 min (≥3 measurements); episodes separated by <15 min are counted as a single event. *p*-values are calculated using the Friedman test for within-participant event counts (*n* = 12, four conditions). CHO, carbohydrate-only snack; CHO + PRO, carbohydrate-plus-protein snack; CHO + FAT, carbohydrate-plus-fat snack; CHO + FIB, carbohydrate-plus-fiber snack.

## Data Availability

The data presented in this study are available from the corresponding author upon reasonable request, subject to ethical and privacy restrictions.

## Supplementary Materials

The following supporting information can be downloaded at: https://www.mdpi.com/article/10.3390/nu18172924/s1, Table S1. Energy and macronutrient composition of the bedtime snack protocols; Table S2. Holm–Bonferroni-adjusted post hoc pairwise comparisons.
