# Supplementary material for: Bedtime Snack Composition and Overnight Glycemic Profiles in Children and Adolescents with Type 1 Diabetes Using Multiple Daily Injections: A Randomized Crossover Study
Source: Nutrients. 2026 Sep 7;18(17):2924. doi: 10.3390/nu18172924 (PMC13567640; doi:10.3390/nu18172924)
Supplement: Supplementary file 1 [file nutrients-18-02924-s001.zip › nutrients-4529614-supplementary.pdf]

**Supplementary Table S1. Energy and macronutrient composition of the bedtime snack protocols**

| Protocol | Food item | Portion size | Carbohydrate, g | Protein, g | Fat, g | Fiber, g | Energy, kcal |
|---|---|---|---|---|---|---|---|
| CHO | Granny Smith apple | 110 g | 14.9 | 0.4 | 0.2 | 3.0 | 64 |
| CHO+PRO | High-protein fresh cheese (140 g SEK Protein Quark + 80 g SEK Strawberry Quark) | 220 g | 15.6 | 20.0 | 4.7 | 0.0 | 190 |
| CHO+FAT | Granny Smith apple + mixed nuts (36 g almonds, 6 g hazelnuts, and 6 g walnuts) | 90 g + 48 g | 15.7 | 11.0 | 25.3 | 7.0 | 350 |
| CHO+FIB | Wasa Fibre whole-grain rye crispbread | 37 g | 15.5 | 4.9 | 2.0 | 10.7 | 122 |

CHO, carbohydrate-only snack; CHO+PRO, carbohydrate-plus-protein snack; CHO+FAT, carbohydrate-plus-fat snack; CHO+FIB, carbohydrate-plus-fiber snack. Macronutrient and energy values were derived from the manufacturers' nutrition labels and BeBiS; fatty acid values were calculated using the manufacturers' nutrition labels and USDA FoodData Central. All snacks were matched for available carbohydrate (approximately 15–16 g) but, by design, were not matched for total energy, which ranged from 64 kcal (CHO) to 350 kcal (CHO+FAT). Fatty acid composition of the fat-containing snacks: CHO+FAT, saturated 2.0 g, monounsaturated 14.6 g, and polyunsaturated 7.7 g; CHO+PRO, saturated 3.1 g, monounsaturated 1.2 g, and polyunsaturated 0.3 g. Note that, for each macronutrient of interest, one protocol provided a high amount and one provided essentially none, whereas the remaining two provided intermediate amounts; between-protocol contrasts therefore reflect the combined effects of whole foods rather than the isolated effect of a single macronutrient.

**Supplementary Table S2.** Holm–Bonferroni-adjusted post hoc pairwise comparisons

| Outcome | CHO vs. CHO+PRO | CHO vs. CHO+FAT | CHO vs. CHO+FIB | CHO+PRO vs. CHO+FAT | CHO+PRO vs. CHO+FIB | CHO+FAT vs. CHO+FIB |
|---|---|---|---|---|---|---|
| TIR, 21:00–09:00; omnibus p = 0.014 | 0.612 | 0.211 | 0.104 | 0.079 | 0.104 | 0.857 |
| TIR, 0–6 h; omnibus p = 0.001 | 0.698 | 0.091 | 0.091 | **0.011** | 0.068 | 0.698 |
| CV, 6–12 h; omnibus p = 0.046 | 0.422 | 0.591 | 0.980 | 0.129 | 0.103 | 0.591 |
| CV, 0–2 h; omnibus p = 0.019 | 0.149 | 0.451 | 0.959 | 0.107 | 0.060 | 0.500 |
| TIR, 2–4 h; omnibus p = 0.008 | 1.000 | 0.124 | 0.109 | 0.091 | 0.124 | 1.000 |
| TIR, 4–6 h; omnibus p = 0.005 | 0.732 | 0.143 | 0.108 | 0.075 | 0.083 | 0.732 |
| Peak-to-nadir glucose amplitude, 21:00–09:00; omnibus p = 0.025 | 0.084 | 0.176 | 0.566 | **0.041** | 0.199 | 0.195 |

Data are Holm–Bonferroni-adjusted p values from pairwise post hoc comparisons performed only for outcomes with a statistically significant omnibus test. CHO, carbohydrate-only snack; CHO+PRO, carbohydrate-plus-protein snack; CHO+FAT, carbohydrate-plus-fat snack; CHO+FIB, carbohydrate-plus-fiber snack; TIR, time in range; CV, coefficient of variation. Bold values indicate statistically significant adjusted p values. Of the seven outcomes with a statistically significant omnibus test, only two pairwise comparisons remained statistically significant after Holm–Bonferroni adjustment, both contrasting CHO+PRO with CHO+FAT (TIR during 0–6 h and peak-to-nadir glucose amplitude). For the remaining outcomes, the omnibus test indicates an overall difference among the four protocols that the present sample size (n = 12) was insufficient to localize to a specific pair of protocols; these nonsignificant adjusted p values should therefore be interpreted as inconclusive (possible Type II error) rather than as evidence of equivalence.
